# Institutionalized Discontent

**DOI:** 10.1007/s12115-016-0033-5

**Published:** 2016-06-14

**Authors:** Neil Gilbert

**Affiliations:** University of California, Berkeley, CA 94720 USA

**Keywords:** Social climate, Sexual violence, Affirmative consent, Saf space, Activism, Campus, Political correctness, Insensitivity, Unatnicipated consequences, Academic mission

## Abstract

Examining past experiences of student activism at Berkeley this article suggests that the present storm of political correctness sweeping American universities represents more than just another of the periodic crusades that have disrupted academic life over the years. The current wave of activism is different largely because the ever-present minorities of militant faculty and student activists have gained significant reinforcements in their struggle to transform the culture and mission of higher education. Over the last several decades federal regulations and funds have created an alternative bureaucracy within universities that is devoted, not to the core academic mission of teaching and research, but to improving the social climate of university life. The legitimacy and power of the social climate bureaucracy depend on heightening the perception that academic life involves a dangerous environment, from which students need protection – a service provided through creating safe spaces, helping students to recognize micro-aggressions, training them in sexual assault prevention, conducting sensitivity training for faculty and the like. Devoted to heightening this perception of the university campus as a hostile environment, the climate bureaucracy has become a source of institutionalized discontent.

Higher education has long been subject to the vagaries of student activism. I will always remember my first day of teaching in 1969 at U.C. Berkeley’s School of Social Welfare. The word had gone out that a new (bearded) Assistant Professor was offering a section of the required research course. Entering the classroom I was greeted by a noisy packed house with people sitting on the floor and the scent of patchouli oil in the air; the tie-dyed attire was colorful, some of the students went bare-footed and by the length of their hair, you knew that the Berkeley barbers had fallen on hard times. As I began to lecture on research design and inferential statistics, a student stood up to inquire, “Hey professor, can you tell us what the fuck has this got to do with the revolution?” Berkeley, after all, was the cradle of free speech. Later I realized that they were probably hoping to have enrolled in a course like one offered by another junior professor in our school, who took his class up to the Berkeley hills where they engaged in water gun fights (as reported in the *Daily Californian*) under the academic guise of learning how challenging it would be to conduct guerilla warfare on this terrain.

The classroom confrontations extended to faculty meetings in the midst of which students would enter uninvited and line up shoulder to shoulder with their backs to the wall – staring with hard-eyes – while their leader made non-negotiable demands banging his fist on the table. At one point there was a rumor that following an example being set on the streets of Oakland some students were planning to bring guns to the school. The faculty felt so threatened that we met off campus in the basement of a colleague’s home. With protestors being fired upon and teargassed at People’s Park, the Black Panthers brandishing arms in Oakland and anti-war demonstrations erupting on many campuses, one had good reasons for apprehension. From classroom demeanor to graduation ceremonies, academic conventions and authority were being defied with impunity. During the early 1970s most social welfare students rejected the bourgeois tradition of caps and gowns at graduation. I recall my first doctoral student came decked out in torn jeans and a black stovepipe hat. Following his unceremonious graduation, Jim Torczyner, went on to become a distinguished professor at McGill University and recipient of many honors including the Jordan Red Crescent’s Gold Medal for Outstanding Achievement in the Humanitarian Field.

At the start of the school term sometime around the mid-1970s, I was stopped in the hallway by one of my colleagues; with raised eyebrows he whispered, “what happened?” Without warning, calm had returned to Haviland Hall, in which our School was housed. Shoes and haircuts were back in fashion as were caps and gowns at graduation. Faculty and students danced and drank together at the annual Christmas party. Beyond the normal conflicts of academic life at Berkeley, things remained generally peaceful in the School of Social Welfare up through the early 1990s, when campuses around the country were unsettled by alarming accounts of an epidemic of sexual assault.

My curiosity about this epidemic was sparked by a news story in the student paper claiming a 25 % rate of rape on the Berkeley campus. This figure seemed surprisingly high in comparison to the crimes on campus reported by the police. Analyzing the Ms. Magazine study on which this claim was based, I published an article in *The Public Interest* suggesting that the epidemic was manufactured by advocacy research that failed to meet the conventional standards of academic rigor and spread by the media’s appetite for sensational headlines.^1^ The many reasons for serious scholars to question the magnitude of rape on campus conveyed by this study include: 73 % of the students categorized as rape victims according to the researcher’s awkward and vaguely worded definition, did not think they had been raped; 42 % of these women had sex again with the man who supposedly raped them; the FBI’s data from 500 colleges and universities with an overall population of 5 million students showed a rate of rape and attempted rape reported to the police which was 1000 times (not percent) smaller than the figure reported in the Ms. Magazine study. It is well-recognized that for many reasons a significant proportion of victims do not make a formal report of their ordeal. But the discrepancy between the FBI’s data and the claims of the Ms. survey is far beyond any reasonable estimate of the magnitude of unreported cases.^2^ The researcher’s assertion that three-quarters of the female students surveyed did not recognize when they had been raped infantilizes college women and in the process trivializes the trauma and pain suffered by rape victims.

A suggestion that advocacy research exaggerates the magnitude of any type of victimization is likely to have unpleasant consequences on and off campus. As Peter Rossi once described in the pages of this journal, his estimate that the size of the homeless population was about ten times smaller than the numbers claimed by the Chicago Coalition for the Homeless resulted in “the longest stretch of personal abuse I have suffered since basic training in the army during World War II”.^3^ In response to my publication criticizing the research cited to fuel anxiety and activism on the Berkeley campus, a vocal minority of the students organized a candlelight vigil; flyers around the campus invited others to join in a demonstration of “student outrage at Professor Gilbert’s article.” By all accounts it was a lively affair during which they marched around campus chanting “Neil Gilbert cut it out or cut it off.” Shortly after, a creepy anonymous threat was slipped under my office door and a student petition was sent to the administration asking them to censure my work.

All this inspired me to delve a little further into the issue. Over the next few years I published several other critical analyses of the rape crisis movement’s advocacy research, including an article in the 1992 issue of *Society*, which was later selected for inclusion in the journals 35th anniversary issue.^4^ As for the student petition, I was confident that the administration had my back and, in fact, never heard a word of it from them. Back then academic freedom to express disruptive views was part of the Berkeley DNA. Judging from my recent experience with the Berkeley administration, today I would probably be reprimanded for “insensitivity” to student concerns. In the 1990s, even the student culture supported the right to express unpopular findings. Though the Daily Californian’s student editorial board did not entirely agree with my work, they “commended Gilbert for publicly challenging the conventional statistics and perceptions of a sensitive issue, in a climate where such alternative viewpoints often receive uncompromising attacks from people who disagree.”^5^ The controversy lingered for a few more years and then student antagonism faded. By 2000 tranquility had again returned to Haviland Hall – and the graduate social welfare student organization voted me the teacher of the year.^6^

## Plus ca change ……Or Is this Time Different?

Recounting my personal experience with the ebb and flow of social activism in the School of Social Welfare during four decades at Berkeley, it is tempting to see the current wave of political correctness as just another periodic tsunami battering the campus rather than a permanent change in the academic climate. In that case, it might be well to simply hunker down and wait for the storm to pass. Yet I have an uneasy feeling that this time campus activism is different. The ever-present minority of vocal student activists and some faculty supporters are not the only agents determined to transform the culture and mission of higher education.

Over the last several decades federal regulations and funds have created an alternative bureaucracy within universities that is devoted, not to the core academic mission of teaching and research, but to improving the social climate of university life. This is an estimable objective in the abstract. And in practice some of the measures taken are of significant benefit; for example, the range of efforts made to accommodate the academic needs of students with disabilities. However, many of the activities promoted by the campus-climate bureaucracy are costly well-intentioned efforts of questionable merit.

Consider, for example, the empirical reality and the policy responses to the renewed panic about sexual assault on campus. According to the FBI’s uniform crime reporting statistics, between 1995 and 2014 the rate of violent crime (which includes murder, forcible rape, robbery, and aggravated assault) in the United States fell by 42 % and the rate of forcible rape declined by 30 %.^7^ The Bureau of Justice Statistics’ annual National Crime Victimization Survey shows that the rate of rape and sexual assault for female college students declined by more than 50 %, from 9.2 per 1000 in 1997 to 4.4 per 1000 in 2013. 8 This number (.0044) is much too small to make a dramatic headline. Since the surveys ask similar questions annually, whatever biases may exist are likely to be constant. Thus, the findings provide a reliable guide to trends in sexual assault over time.

Despite the significant increase in public safety, campus life in America continues to be depicted as alarmingly dangerous. So dangerous in fact that it is hard to imagine why any parents who believed the widespread claims of sexual assault on campus would pay $40,000–50,000 a year to send their daughters to college. A New York Times headline declares, “1 in 4 Women Experience Sex Assault on Campus,” according to findings from a 2015 survey commissioned by the American Association of Universities (AAU).^9^ This often-quoted statistic seems to bolster the 25 % rate of rape on campus claimed 25 years earlier, except for the fact that the current figure is based on a definition of sexual assault that includes, forced kissing, touching, and rubbing up against someone in a sexual way, even if it’s over their clothes. Conflating an unwanted kiss or dancing too closely with forcible rape inflates the rate while it dilutes the meaning of sexual violence.

Why have the conceptual boundaries of sexual violence on campus expanded, as the depth of the problem has diminished throughout the nation? One explanation, as Irving Kristol observed, is that it’s a matter “of jobs, status, and power”.^10^ To increase the social climate bureaucracy’s jobs, status and power, it must be shown that serious social problems exist on campus creating a hostile environment. Hence, this bureaucracy is perforce an embedded a source of institutionalized discontent; its existence is justified less by promoting the university’s core mission than by drawing attention to the alleged magnitude of social troubles on campus and the number of victims that need to be served. A perception reinforced by the media’s inclination to publicize research claiming that problems such as sexual violence on campus are rampant, while ignoring the definitions, measurements, and response rates on which these claims are based.^11^

The AAU study that reported one in four college women as victims of sexual assault is a case in point. This widely-cited figure is based on an online survey which had a response rate of only 19.3 %. The study’s authors admit that the findings exaggerate the degree of victimization, since there was evidence that non-respondents were less likely to report being victims. How much these findings are biased is anyone’s guess. The biased sample aside, looking at the definition of sexual assault in this study we find that 57 % of these cases involved misconduct in the category of sexual touching, which included unwanted kisses. It is not surprising that when asked why they had not reported these incidents, 75 % of the women identified as victims responded, “I did not think it was serious enough to report.” What is surprising, however, is that almost 60 % of those identified as victims of forced penetration, also said they “did not think it was serious enough to report.” Even more curious, perhaps, is the fact that when asked: “How likely do you think it is that *you* will experience sexual assault or sexual misconduct on campus?” only 8 % of the women thought that it was “very” or “extremely” likely – even though according to the survey 25 % already had been victims of sexual assault and almost 60 % victims of sexual misconduct.^12^ These incongruities underscore just how difficult it is to get a clear understanding of the scope and personal experience of sexual misconduct on college campuses through student surveys. Different pictures emerge from these surveys depending on how the questions are phrased and sequenced, the researcher’s definitions, response rates and how the findings are reported.

One thing is clear, the alarming rate of sexual assaults on campus claimed by some studies such as the AAU survey does not bear the faintest resemblance to the palpable level of victimization reported by students on the ground. According to the California State Auditor’s report from 2009 through 2013 there was a total of 241 student-related sexual harassment or sexual violence complaints made to the campus authorities at the University of California, Berkeley. That is an average of 48 complaints a year on a campus with an enrollment of more than 18,000 women. (A similar rate was reported on the UCLA campus.) Moreover, the auditor’s report indicates that a significant number of these cases may be duplicative as, “for example, the 49 complaints in the University of California Berkeley’s student conduct office may be included in the 120 total complaints tallied by its Title IX office”.^13^ And it should be recognized that these complaints were not all verified. From 2008 through 2014, for example, 76 complaints of sexual misconduct were resolved by the U.C. Berkeley Center on Student Conduct; in 22 % of the cases the allegations were not verified; the vast majority of the 76 cases involved sexual harassment/stalking; non-consensual sexual intercourse averaged less than two cases annually.^14^

If we accept the California State Auditor’s overall figures (leaving aside that some of the cases may be duplicative), the annual average of 48 complaints of sexual misconduct reported on the Berkeley campus translates to a rate of 2.5 cases per 1000 female students, which is relatively small compared to various survey findings. One explanation, of course, is that when behaviors such as touching and unwanted kisses are included, many instances of sexual misconduct go unreported. Still 48 reported cases a year involves a substantial number of potential victims and should be taken very seriously. In fact considerable resources are devoted to providing a safe supportive atmosphere for reporting incidents as well as professional services to deal with these cases. There are six different offices on the Berkeley campus that are officially designated to receive reports of sexual misconduct and at least 18 mental-health professionals, available to assist victims of sexual misconduct. These positions do not include four counsellors in the student health center who work with victims of sexual violence, administrative assistants or any of the relevant personnel in the campus police department.

Moreover, in May 2016 the Berkeley administration announced plans to hire ten additional social climate professionals to deal with sexual violence on campus. Just for a sense of proportion, consider that in 2007 public prosecutors throughout the United States carried an average of 94 felony cases per attorney.^15^ Unlike sexual harassment, fondling and unwanted kisses, which represent many if not most of the cases reported on campus, felonies are crimes including murder and rape, which are punishable by imprisonment for more than one year or death. If the 10 additional staff had to work in the real world of criminal violence they would handle on average a total of 940 cases a year, compared to the average of 48 cases of sexual misconduct reported at Berkeley over the last several years.

The resources devoted to the problem of sexual violence on campus appear to be quite high compared not only to the number of cases reported annually but also to resources available in many other communities where the risks of sexual violence are greater. Although poor and minority women are more likely to be victims of rape than middle-class college students, their communities typically receive considerably less public support for counselling centers and prevention programs.^16^

In addition to investigating sexual misconduct complaints and providing supportive services for victims, the non-academic staff are also engaged in prevention training. Every student coming to Berkeley is required to complete an in-person course and an on-line module on sexual assault education. There is not a scintilla of evidence that this training has any impact on the rate of sexual misconduct. Yet, those who do not complete these requirements have their registration blocked, a penalty more severe than any imposed for failure of an academic course. This symbolizes the remarkable shift in the balance of power between the university’s academic mission and the social climate bureaucracy’s agenda, which is seen even more clearly in the allocation of resources. Between 2000 and 2015 the number of full-time ladder-rank teaching faculty at Berkeley increased by 1 % while the number of full-time staff providing student services and health care increased by more than 100 %, at which point they outnumbered the teaching faculty by 13 %.^17^

Faculty are also required to participate in “sexual harassment and sexual violence prevention and awareness training.” Unlike students who can evidently retain whatever they learn from one course, faculty are obliged to repeat their training annually. Many faculty not just at Berkeley, but across the country, consider this periodic genuflecting to the campus climate bureaucracy a serious waste of time. Laura Kipnis describes her reaction to the list of guidelines handed out in a faculty sexual harassment workshop at Northwestern University. The first imperative was “Do not make unwanted sexual advances.” Never hesitant to voice the politically incorrect thought on everyone’s mind, Kipnis inquired, “But how do you know they’re unwanted until you try?”.^18^

## Managing Behavior: When to Speak and What to Say

One of the important lessons conveyed in sexual assault education involves the recent directive that requires ongoing *affirmative consent* to be expressed in all sexual activity every step of the way from kissing to touching to whatever comes after that, regardless of how long and how intimate the relationship between the parties involved. This literally means that vocal permission must be asked and received for two students to kiss goodbye after spending a weekend in bed together. In the absence of affirmative consent, the goodbye kiss is deemed as sexual misconduct.^19^ According to the new directive, spontaneity is too dangerous as one of the parties may have gotten the wrong signal. Silence cannot be taken as consent.

This bureaucratic effort to micromanage intimate relations among young adults is lampooned in popular programs such as South Park, which amuses millions of young viewers with the antics of P.C. Principal knocking on the bedroom doors of a fraternity house to collect signed affirmative consent forms that detail the occupants’ explicit sexual activities. Although it is unlikely to increase anyone’s safety, the latest bid to regulate sexual encounters does have an effect on some students as I learned through a minor incident during one my lectures.^20^ At the start of the class several questions were raised about an upcoming mid-term exam. After a few minutes of discussion I asked if anyone had other questions, before moving on. There was no answer so without thinking I said “ok I’ll take your silence as consent” and began the days lecture. During the break a student came over to tell me that a number of her classmates were “offended” by my insensitive comment. Later one of the offended parties e-mailed a note to explain that I had not taken into account “the psychological effect that priming” from various sources, including the graduate-wide sexual assault training, had on their readiness to take a common phrase out of context and imagine that it alluded to sexual relations. Only a minority of students explicitly expressed this response and several others indicated that they found nothing offensive in the “consent” comment.

That small incident, however, is indicative of the social climate bureaucracy’s larger impact on the psychological environment of university life. According to the established narrative the dangers of campus life go well beyond the alleged epidemic of sexual assault. The pervasive sense of danger is conveyed in the Newspeak of safe spaces, trigger warnings, brave conversations, and reparative apologies – all referring to the psychic threats lurking and potential harms inflicted in what was heretofore considered normal academic discourse.

The familiar response has been a demand to create “safe spaces” in which students may engage in challenging conversations. The normal academic classroom is evidently no longer such a place or at the very least faculty and students now need special training in order to make classrooms safe for the discussion of difficult topics. For the first time in its history, such training was provided at the beginning of the academic year for all in-coming students and all the teaching staff at Berkeley Social Welfare. An outside consultant planned the agenda and facilitated the day’s activities, which were designed to increase self-awareness and social sensitivity regarding matters such as privilege, power, racism and oppression. Some participants enjoyed the experience and found it useful. Others failed to see any significant value for the time invested.

For me, it was a day awash in platitudes. We were introduced to principles for conducting dialogues, such as “find common ground,” “listen without judgement,” “work toward understanding’ and “there is no right and wrong.” There was a lesson to raise our awareness of privilege. Having achieved this heightened sensitivity, it was not clear what we were supposed to do except, perhaps, feel guilty.^21^ In a lecture on facilitating difficult conversations about racism and oppression we were advised not to stand during the discussion and if “conflict arises try making yourself small. Crouch down or *drop on one knee if you are physically able*.” 22 A group exercise on communication skills, had about twenty faculty standing in a circle calling each other’s names and throwing around invisible “colored” balls for ten minutes to learn that too much noise hinders communications. Watching my colleagues in a circle throwing around invisible balls, I could not help thinking about how much money a full day of our entire faculty’s time, outside consultant fees and lunch for over 100 participants was costing the university – and how much it would cost if every department on campus provided this training. Arguably, social welfare graduate students are among those on campus least in need of sensitivity training on these matters.

During the Spring semester a follow-up workshop with the outside consultants was conducted for all the students, which required the cancellation of graduate classes for half a day. The faculty did not object. Some probably felt it imprudent to question the administration’s request or express reservations about the student’s need for another workshop. Anyway it was one less class to teach. This training session, of course, could have been scheduled sometime over a weekend or during the Spring break. Even on a school day the vast majority of students, quietly voting with their feet, did not attend the workshop.^23^ However, the ease with which it displaced regularly scheduled classes is yet another example of the extent to which social climate concerns have taken precedence over the academic mission.

## Academic Climate Change: A Chilling Effect

The social climate bureaucracy is devoted to increasing sensitivity, eliminating micro-aggressions, relieving potential sources of stress and in other ways creating a warm welcoming safe environment. However, greeting in-coming students with information that sexual violence is so pervasive that they must take training in sexually assault prevention before they can even register for courses is likely to heighten anxieties among some of the young newcomers to the campus. One might justify this chilling welcome if the prevailing assumptions about the epidemic of sexual violence on campus were indeed valid and if the training actually could be shown to make any difference in protecting students against sexual misconduct. In neither case is there any convincing evidence to support these assertions. The students’ first intellectual experience with in-person instruction on the Berkeley campus is not with Shakespeare, Hamilton, Angelou, Baldwin, Currie, Hayek or Marx but with how to immunize themselves against an alleged epidemic of sexual assault.

And there are other unanticipated consequences. Striving to ease the psychological strain of coming to grips with provocative ideas and discomforting topics, the various deliberations devoted to trigger warnings, micro-aggressions and safe spaces may very well exacerbate the tensions they seek to alleviate – or impel faculty to avoid challenging topics. Some students now come to class primed to detect micro-aggressions, alert to the heresy of insensitivity, and licensed to find disagreeable views and phrases personally oppressive. To be sure, most students are neither so motivated nor so easily offended. But it only takes a vocal minority to cast a chill over classroom interaction, particularly when an administration feels the need to appease the few who take offense. Greg Lukianoff and Jonathan Haidt tell of seven humanities professors who report the “chilling effect” of the demand for trigger warnings on their teaching, in an academic atmosphere where deans and administrators were calling faculty in response to student complaints about discomforting material presented in their classes. “When students come to *expect* trigger warnings for any material that makes them uncomfortable, the easiest way for faculty to stay out of trouble is to avoid material that might upset the most sensitive student in the class.” 24 And that is in the humanities. Imagine a course in social welfare policy that might cover child abuse, rape, racism, abortion, divorce, single-parenthood, and mental illness.

Beyond inhibiting spontaneity, humor and controversial ideas, the social climate bureaucracy is costly. On the Berkeley campus the costs include not only outside consultants’ fees and faculty time, but the addition of 700 professional staff offering student services hired over the last 15 years, during which time only 70 positions were added to the rooster of full-time ladder-rank academic faculty. The time when the State paid for all this is long past. Berkeley is currently running an unprecedented structural deficit of roughly $150 million. Academic units have already been informed to anticipate being downsized. As austerity measures are applied we would expect tensions to heat up between academic interests dedicated to advancing the university’s core mission and bureaucratic commitments to improving the social climate. While the latter have a vocal constituency of students and activist faculty, with few exceptions the former have been muted. And those exceptions involve several high profile cases in which faculty and administrators suffered personal attacks and came under pressure to resign, which has made others think twice about coming forward. Many faculty and students are troubled by the current course of events on campus but hesitant to stand up and voice support for the academic mission of university life. They would do well to recall the Latin proverb “qui tacet consentit – silence gives consent.

## Footnotes

1 Neil Gilbert, “The Phantom Epidemic of Sexual Assault,” *The Public Interest*. (Spring 1991), pp.54–65.).

2 The U.S. Bureau of Justice statistics estimates that the victimization rate of rape and sexual assault for students is five times higher than reported to the police. Sofi Sinozich and Lynn Langton, “Rape and Sexual Assault Victimization Among College-Age Females, 1995–2013,” Bureau of Justice Statistics, December 2014.

3 Peter H. Rossi, “ No Good Applied Social Research Goes Unpunished,” *Society*, 25:1 (1987), pp.73–79.

4 Neil Gilbert. “Realities and Mythologies of Rape,” *Society* (May/June, 1992); Neil Gilbert Neil Gilbert. “Advocacy Research and Social Policy,” in Michael Tonry (Ed.), *Crime and Justice*: *A Review of Research* (Chicago: University of Chicago Press, 1997); Neil Gilbert, “The Campus Rape Scare,” *Wall Street Journal*, June 27, 1991, p. A14.

5 Editorial, *The Daily Californian*, Friday June 21,1991, p.4.

6 I was the only non-minority male professor in the School of Social Welfare to have received such an honor.

7 FBI, Crime in the United States, 2014 https://www.fbi.gov/about-us/cjis/ucr/crime-in-the-u.s/2014/crime-in-the-u.s.-2014/tables/table-1

8 Sofi Sinozich and Lynn Langton, *Rape and Sexual Assault Victimization Among College-Age Females, 1995–2013* U.S. Department of Justice Office of Justice Programs Bureau of Justice Statistics (December 2014) http://www.bjs.gov/content/pub/pdf/rsavcaf9513.pdf Accessed 3/9/16

9 Richard Perez-Pena,  “1 in 4 Women Experience Sex Assault on Campus, “ New York Times September 22, 1015, p.A17. Similar coverage was given by the *Washington Post*, *The Huffington Post* and *Slate*, to name only a few.

10 Irving Kristol,“Taxes, poverty and equality,” *The Public Interest,* 37 (Fall 1974) p.28.

11 Joel Best, “Promoting Bad Statistics,” *Society* 38:3 (March/April 2001), pp. 10--15.

12 David Cantor, Bonnie Fisher, Susan Chibnall, Reanne Townsend, Hyunshik Lee, Carol Bruce, Gail, *Thomas Report on the AAU Campus Climate Survey on Sexual Assault and Sexual Misconduct* Prepared by Westat for The Association of American Universities (Sept.21, 2015) https://www.aau.edu/uploadedFiles/AAU_Publications/AAU_Reports/Sexual_Assault_Campus_Survey/AAU_Campus_Climate_Survey_12_14_15.pdf accessed 3/7/16.

13 California State Auditor, *Sexual Harassment and Sexual Violence Report 2013-124* (June 2014)., p.67.

14 UC Berkeley Center for Student Misconduct Sexual Misconduct Cases 2008–2014. http://sexualassault.berkeley.edu/wp-content/uploads/2015/01/CSCDataforCALegislature1.7.15.pdf accessed 3/11/16

15 U.S. Department of Justice, Bureau of Justice Statistics, 2007 National Census of State Court Prosecutors – Statistical files December 2011. http://www.bjs.gov/content/pub/ascii/psc07st.txt

16 This is highlighted in the award winning series by Nara Schoenberg and Sam Roe, “Rape: The Making of an Epidemic,” *The Toledo Blade*, October 10, 11, 12, 1993.

17 University of California Office of the President, Statistical Summary and Data on UC Students, Faculty, and Staff http://legacy-its.ucop.edu/uwnews/stat/

18 Laura Kipnis,“Sexual Paranoia Strikes Academe,” *The Chronicle of Higher Education –The Chronicle Review,* February 27, 2015. http://chronicle.com/article/Sexual-Paranoia-Strikes/190351/

19 David Bernstein , “YOU are a rapist; yes YOU!” *Washington Post,* June 23, 2014 https://www.washingtonpost.com/news/volokh-conspiracy/wp/2014/06/23/you-are-a-rapist-yes-you/ accessed 3/11/16

20 This was a graduate course in which the students were on average in their late-twenties.

21 I thought I probably just did not get it, until I read John McWhorter’s insightful essay questioning from a minority person’s perspective what these lessons on White privilege were really trying to achieve and for whose benefit. John McWhorter, “The Privilege of Checking White Privilege,” *The Daily Beast*http://www.thedailybeast.com/articles/2015/03/15/the-privilege-of-checking-white-privilege.html

22 inVision Consulting 2015, “Engaging Compassionate Conversations on Racism and Systemic Oppression.” Presented at the Berkeley School of Social Welfare New Student Orientation, 08/26/2015.

23 Only 25 % of those invited to the workshop were in attendance by the end of the first hour.

24 Greg Lukianoff and Jonathan Haidt, “The Coddling of the American Mind,” *The Atlantic*, 316:2 (September 2015), p.49.

